# Supplementary material for: Efficient Quasi-2D Perovskite Light-Emitting Diodes Enabled by Regulating Phase Distribution with a Fluorinated Organic Cation
Source: Nanomaterials (Basel). 2022 Oct 6;12(19):3495. doi: 10.3390/nano12193495 (PMC9565347; doi:10.3390/nano12193495)
Supplement: Supplementary file 1 [file nanomaterials-12-03495-s001.zip › nanomaterials-1909773-supplementary.pdf]

nanomaterials

MDPI

Supplementary Materials: Efficient Quasi-2D Perovskite Light-Emitting Diodes Enabled by Regulating Phase Distribution with a Fluorinated Organic Cation

Ziqing Ye [1], Junmin Xia [2], Dengliang Zhang [1], Xingxing Duan [1], Zhaohui Xing [1], Guangrong Jin [1], Yongqing Cai [2], Guichuan Xing [2,*], Jiangshan Chen [1,*] and Dongge Ma [1]

[1] Institute of Polymer Optoelectronic Materials and Devices, State Key Laboratory of Luminescent Materials and Devices, Guangdong Provincial Key Laboratory of Luminescence from Molecular Aggregates, South China University of Technology, Guangzhou 510640, China

[2] Joint Key Laboratory of the Ministry of Education, Institute of Applied Physics and Materials Engineering, University of Macau, Macau 999078, China

* Correspondence: gcxing@um.edu.mo (G.X.); msjschen@scut.edu.cn (J.C.)

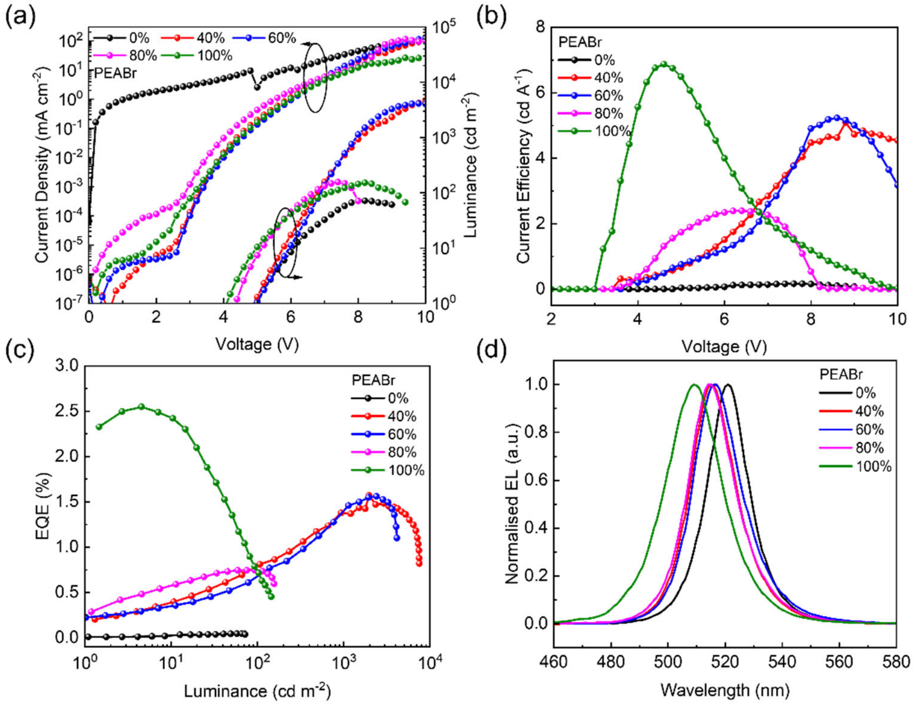

Figure S1. Performance of the quasi-2D PeLEDs based on different contents of PEABr at the annealing temperature of 110 °C: (**a**) Current density-voltage-luminance (J-V-L) characteristics; (**b**) Current efficiency-voltage (CE-V) characteristics; (**c**) External quantum efficiency-luminance (EQE-L) characteristics; and (**d**) Electroluminescence (EL) spectra at 6V.

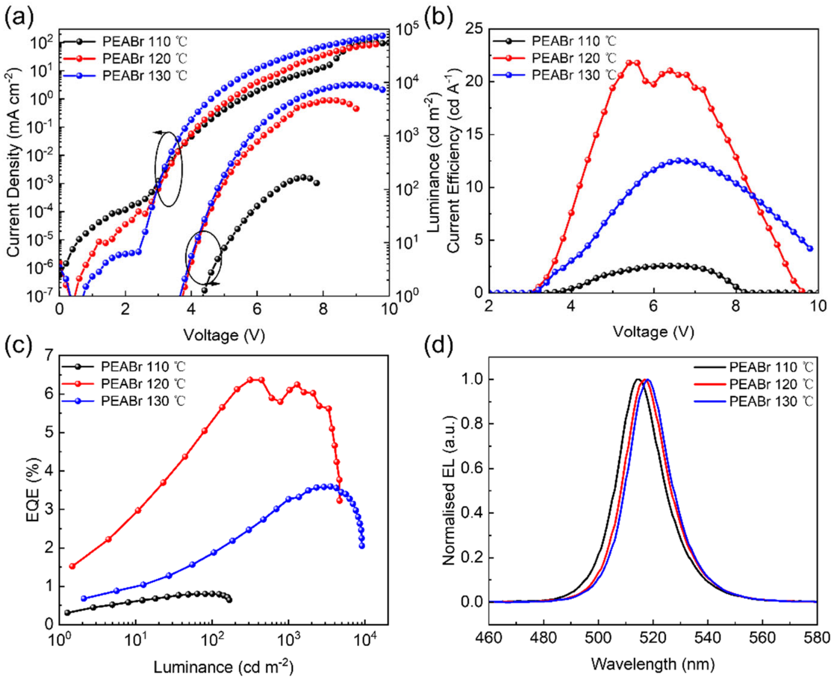

Figure S2. Performance of the quasi-2D PeLEDs based on 80% PEABr at different annealing temperatures: (**a**) J-V-L characteristics; (**b**) CE-V characteristics; (**c**) EQE-L characteristics; and (**d**) EL spectra at 6V bias voltage.

Table S1. Performance of the quasi-2D PeLEDs based on different contents of PEABr and *m*-FPEABr at the annealing temperature of 110 °C.

Organic Cations	Content (%)	V_{on} [a] (V)	L_{max} [b] (cd m^{-2})	CE_{max} [c] (cd A^{-1})	EQE_{max} [d] (%)	λ_{max} [e] (nm)
w/o	0	5.0	72	0.17	0.05	521
PEABr	40	5.0	7535	5.1	1.57	515
	60	5.0	4127	5.23	1.56	517
	80	4.4	168	2.58	0.80	514
	100	4.2	145	6.88	2.55	509
m-FPEABr	40	3.8	10007	4.49	1.38	515
	60	3.2	11005	26.71	8.53	514
	80	3.2	10603	36.05	11.41	514
	100	3.4	1217	21.39	6.70	515

[a] Turn-on voltage. [b] Maximum luminance. [c] Maximum CE. [d] Maximum EQE. [e] EL peak.

Table S2. Performance of the quasi-2D PeLEDs based on PEABr and *m*-FPEABr at different annealing temperatures.

Organic Cations	Temperature (°C)	V_{on} (V)	L_{max} (cd m^{-2})	CE_{max} (cd A^{-1})	EQE_{max} (%)	λ_{max} (nm)
PEABr	110	4.4	168	2.58	0.80	514
	120	4.2	4650	21.03	6.25	517
	130	3.8	9115	12.49	3.59	518
m-FPEABr	90	3.4	5367	19.62	6.10	515
	110	3.2	10603	36.05	11.41	514
	120	3.4	10532	54.95	16.66	515
	130	3.4	18660	34.52	9.95	518
	140	3.4	28039	23.25	6.55	519

We obtained the time-resolved photoluminescence (TRPL) spectra of the two perovskites by time-correlated single photon counting (TCSPC) spectroscopy. Time constant and amplitude of perovskite films can be obtained by triple-exponential fitting to the corresponding PL decay curves, as shown in Equations (S1), (S2) and (S3):

$$I = \sum B_{\mathrm{i}}\, e^{\frac{-t}{\tau_{\mathrm{i}}}}, i = 1, 2 \text{ and } 3 \quad \text{(S1)}$$

$$\tau_{ave} = \frac{\Sigma B_i \tau_i^2}{\Sigma B_i \tau_i}, i = 1, 2 \text{ and } 3 \quad \text{(S2)}$$

$$A_i = \frac{A_i \tau_i}{\Sigma A_i \tau_i}, i = 1, 2 \text{ and } 3 \quad \text{(S3)}$$

where I is the normalized fluorescence intensity; t is time; A_1, A_2 and A_3 are relative amplitude constants; B_1, B_2 and B_3 are amplitude constants; τ_1, τ_2 and τ_3 represent the decay lifetimes of carriers; and τ_{ave} represents the average lifetime of carriers.

The corresponding time constants and amplitudes for the PEA and *m*-FPEAperovskites have been listed as follows:

Table S3. Exponential fits for PL decay curves of the PEABr and *m*-FPEABr perovskites.

**Sample**	**A_1**	**τ_1 (ns)**	**A_2**	**τ_2 (ns)**	**A_3**	**τ_3 (ns)**	**τ_{ave} (ns)**
PEABr	0.27	6.50	0.51	34.27	0.23	244.61	74.91
m-FPEABr	0.18	7.37	0.43	47.05	0.39	307.28	141.83

The kinetics of the ground state bleaching (GSB) are fitted by a multiple-exponential function as Formula (S4):

$$\Delta A = \sum_{n=i} a_i\, e^{\left(-\frac{t}{\tau_i}\right)} - c_1 e^{\left(-\frac{t}{\tau_r}\right)}, i = 1, 2, 3 \ldots \quad \text{(S4)}$$

where a_1, a_2, a_3 and c_1 are amplitudes; τ_1, τ_2 and τ_3 are decay time constants; τ_r is formation time constant. According to the actual situation, a four-exponential fit was used for the low-n phases with n = 2, 3 and 4, and a tri-exponential fit was used for the high-n phases with n ≥ 5, and the results have been listed as follows [1–3]:

Table S4. TA carrier dynamics fitting results of the PEABr and *m*-FPEABr perovskites.

**Sample**	**GSB**	**τ_r (ps)**	**τ_1 (ps)**	**τ_2 (ps)**	**τ_3 (ps)**
**PEABr**	n≥5	0.3	113.5	2989	
m-FPEABr	n=2	0.11	0.23	7.12	881
	n=3	0.18	0.31	5.42	212
	n=4	0.27	0.30	6.80	147
	n≥5	0.38	91.76	3607	

The T = 0 K formation enthalpies are estimated using density functional theory (DFT).We calculated total energies as implemented in VASP with the spin-polarized generalized gradient approximation (GGA) proposed by Perdew–Burke–Ernzerhof (PBE) [4]. The structure relaxations are carried out with a 400eV plane-wave cutoff, and the van der Waals force correction is considered with the DFT-D3 method of Grimme [5]. The Brillouin Zone integration was sampled following a gamma-centered Monkhorst-pack scheme, using 2 × 2× 2 k-point grids [6]. The total energy tolerance for the electronic energy minimization was 10^{-4} eV; for structure optimization, forces were minimized such that all atoms experience forces <0.01eVÅ^{-1} after relaxation of the ionic coordinates and unit cell shape and volume, respectively.

The formation energies were calculated with the following equation:

$$\Delta H_f = E_{2D} - 2E_{ABr} - E_{PbBr_2} \quad \text{(S5)}$$

Here, E_f is the formation energy, while E_{2D} is the energy of the 2D perovskite, E_{ABr} and E_{PbBr2} are the energy of the relative phenylethylammonium-bromide salts (ABr, A = PEA and *m*-FPEA) and the $PbBr_2$. The structures of fluorinated phenylethylammonium-bromide salts used for formation enthalpy calculations are obtained by relaxation of the reported structure of phenylethylammonium bromide, after substitution of the relevant hydrogen with fluorine. The structures of fluorinated 2D perovskites are obtained by relaxation of the reported structure of $mF1PEA_2PbI_4$ reported formerly, after substitution of the iodine by bromine [7,8].

Table S5. Calculated formation enthalpy of PEA_2PbBr_4 and *m*-$FPEA_2PbBr_4$ 2D perovskites.

Perovskite	PEA_2PbBr_4	*m*-$FPEA_2PbBr_4$
ΔH_f^{0K} (eV/formula unit)	−0.48	−0.76

References

1. Guo, Z.; Zhang, Y.; Wang, B.; Wang, L.; Zhou, N.; Qiu, Z.; Li, N.; Chen, Y.; Zhu, C.; Xie, H.; et al. Promoting Energy Transfer via Manipulation of Crystallization Kinetics of Quasi-2D Perovskites for Efficient Green Light-Emitting Diodes. *Adv. Mater.* **2021**, *33*, 2102246, doi:10.1002/adma.202102246.
2. Li, M.; Gao, Q.; Liu, P.; Liao, Q.; Zhang, H.; Yao, J.; Hu, W.; Wu, Y.; Fu, H. Amplified Spontaneous Emission Based on 2D Ruddlesden-Popper Perovskites. *Adv. Funct. Mater.* **2018**, *28*, 1707006, doi:10.1002/adfm.201707006.
3. Cho, H.; Jeong, S.-H.; Park, M.-H.; Kim, Y.-H.; Wolf, C.; Lee, C.-L.; Heo, J.H.; Sadhanala, A.; Myoung, N.; Yoo, S.; et al. Overcoming the Electroluminescence Efficiency Limitations of Perovskite Light-Emitting Diodes. *Science* **2015**, *350*, 1222–1225, doi:10.1126/science.aad1818.
4. Perdew, J.P.; Burke, K.; Ernzerhof, M. Generalized Gradient Approximation Made Simple. *Phys. Rev. Lett.* **1996**, *77*, 3865–3868, doi:10.1103/PhysRevLett.77.3865.
5. Kresse, G.; Joubert, D. From Ultrasoft Pseudopotentials to the Projector Augmented-Wave Method. *Phys. Rev. B* **1999**, *59*, 1758–1775, doi:10.1103/PhysRevB.59.1758.
6. Monkhorst, H.J.; Pack, J.D. Special Points for Brillouin-Zone Integrations. *Phys. Rev. B* **1976**, *13*, 5188–5192, doi:10.1103/PhysRevB.13.5188.
7. Hu, J.; Oswald, I.W.H.H.; Stuard, S.J.; Nahid, M.M.; Zhou, N.; Williams, O.F.; Guo, Z.; Yan, L.; Hu, H.; Chen, Z.; et al. Synthetic Control over Orientational Degeneracy of Spacer Cations Enhances Solar Cell Efficiency in Two-Dimensional Perovskites. *Nat. Commun.* **2019**, *10*, 1276–1286, doi:10.1038/s41467-019-08980-x.
8. Rademeyer, M. 2-Phenylethylammonium Bromide. *Acta Crystallogr., Sect. E: Struct. Rep. Online* **2007**, *63*, o221–o223, doi:10.1107/S1600536806051488.
